# Two Cases of Bilateral Rhegmatogenous Retinal Detachment During Orthokeratology Treatment

**DOI:** 10.7759/cureus.50958

**Published:** 2023-12-22

**Authors:** Miyo Yoshida, Kosei Tomita, Masayuki Akimoto

**Affiliations:** 1 Ophthalmology, Osaka Red Cross Hospital, Osaka, JPN

**Keywords:** surgical intervention, high myopia, young children, rhegmatogenous retinal detachment, orthokeratology

## Abstract

Orthokeratology may be effective in slowing myopic progression. However, whether orthokeratology is beneficial enough to prevent rhegmatogenous retinal detachment formation remains unclear. Two cases of bilateral rhegmatogenous retinal detachment were seen during orthokeratology treatment and corrected with scleral buckling and cryopexy under general anesthesia. This is the first report of bilateral retinal detachment found during orthokeratology treatment. Although orthokeratology is effective for myopic correction and prevents axial length elongation, patients still have a risk of rhegmatogenous retinal detachment. Careful follow-up not only of the anterior segment but also of the peripheral retina is necessary.

## Introduction

Orthokeratology is a method of correcting myopia in which a rigid contact lens is worn at bedtime to temporarily change the corneal shape and provide corrected vision during the day without the use of contact lenses or glasses [1-5]. Some reports suggest that elongation of the axial length can be prevented [1-3], and its use is increasing globally [6,7]. Pathological myopia is a risk for blinding diseases such as retinal detachment and myopic macular disease [8,9], and improvement of axial myopia is expected to lower those risks. Despite the benefits of orthokeratology, numerous reports have been published regarding sight-threatening complications, such as contact lens infections, corneal erosions, and scars [4,5]. These complications are associated with overnight contact lens use. However, there have been no reports of other ocular complications such as ocular fundus disease. Here, our recent experience is reported treating two boys with bilateral rhegmatogenous retinal detachment (RRD), which was found while they were undergoing orthokeratology treatment.

## Case presentation

Case 1

**Figure 1:**
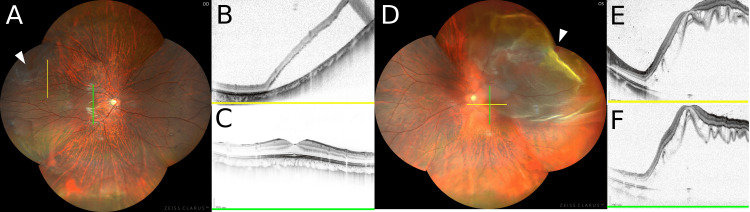
Case 1 A. Panoramic fundus photograph of the right eye. The section of the optical coherence tomography (OCT) image is indicated (lines). The arrowhead indicates the retinal hole. B.  OCT image of the right eye (yellow line). C. OCT image of the macular in the right eye (green line). D. Panoramic fundus photograph of the left eye. The section of OCT is indicated (lines). The arrowhead indicates the retinal hole. E. OCT image of the macular in the left eye (yellow line). F. OCT image of the macular in the left eye (green line).

A 16-year-old boy presented to our clinic with an acute visual field defect in the left eye. He had undergone orthokeratology for myopic control for nine years and saw his orthokeratology specialist for regular check-ups every three months. He had no history of trauma or ocular or systemic disease. He visited the clinic after noticing vision loss and visual field defects in his left eye and was referred to our hospital after being diagnosed with a detached retina in the left eye. At the initial visit, the best-corrected visual acuity (BCVA) (logarithm of minimal angle of resolution) was -0.1 and 1.0 in the right and left eyes, respectively. His manifest refractive error in the right eye was -0.25 spherical diopters and astigmatism of -0.50 cylinder in the axis 25^o^, and in the left eye was not corrigible. Cycloplegic refraction was not checked. The axial lengths were 27.26 mm in the right eye and 27.23 mm in the left eye. The intraocular pressure (IOP) was 15 mmHg in the right eye and 13 mmHg in the left eye. Slit-lamp biomicroscopy showed that the anterior chamber and crystalline lens of both eyes were normal, but ophthalmoscopy showed a superior RRD with macular detachment involving three retinal holes and multiple lattice degenerations in three quadrants in the left eye (Figures 1D-1F). Surprisingly, in the right eye, there was also a supra-temporal RRD with no macular detachment with a retinal hole (Figures 1A-1C). Segmental scleral buckling surgery for the right eye and encircling circumferential scleral buckling of the left eye was performed under general anesthesia. No intraoperative or postoperative complications were encountered. Retinal reattachments were achieved in both eyes, and BCVA improved to -0.2 and -0.1 in the right and left eyes, respectively. Because of the complete return to baseline refraction after discontinuation of corneal refractive therapy and the change of postoperative vitreous cavity length, his manifest refractive error in the right eye was -7.00 spherical diopters and astigmatism of -1.50 cylinder in the axis 180^o^, and in the left eye was -7.50 spherical diopters and astigmatism of -1.50 cylinder in the axis 180^o^ at three months postoperatively.

Case 2

**Figure 2:**
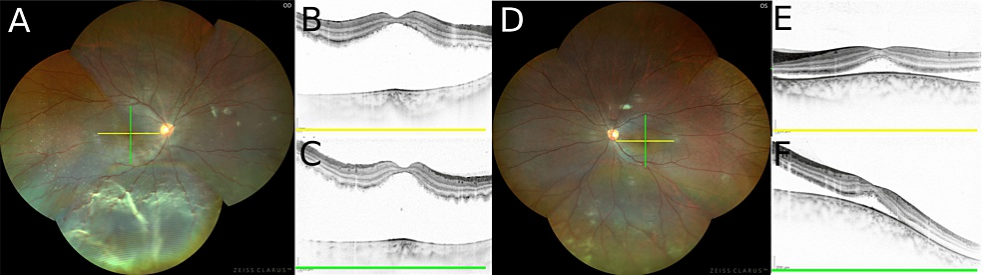
Case 2 A.  Panoramic fundus photograph of the right eye. The section of the optical coherence tomography (OCT) image is indicated (lines). B.  OCT image of the macular in the right eye (yellow line). C.  OCT image of the macular in the right eye (green line). D.  Panoramic fundus photograph of the left eye. The section of OCT image is indicated (lines). E.  OCT image of the macular in the left eye (yellow line). F.  OCT image of the macular in the left eye (green line).

 A nine-year-old boy presented to our hospital with instability in visual acuity in both eyes for four months. He had undergone orthokeratology in the left eye for three years and in the right eye for six months. He did not have any history of trauma or ocular or systemic diseases as in case 1. At the first visit, the BCVA was 0.4 and 0.1 in the right and left eyes, respectively. The manifest refractive error in the right eye was -2.00 spherical diopters and astigmatism of -1.50 cylinder in the axis 37^o^, and in the left eye was -3.00 spherical diopters and astigmatism of -0.50 cylinder in the axis 161^o^. Cycloplegic refraction was not checked. The axial lengths were 23.91 mm in the right eye and 25.15 mm in the left eye. The IOP was 7 mm Hg in the right eye and 14 mm Hg in the left eye. Although the anterior chamber and crystalline lens of both eyes were normal, ophthalmoscopy showed an RRD with macular detachment and one retinal break in both eyes (Figures 2A-2F). Encircling circumferential scleral buckling of both eyes was performed. No intraoperative or postoperative complications were noted. Retinal reattachments were achieved in both eyes, and BCVA slightly improved to 0.2 and 0.1 in the right and left eyes, respectively, at six months postoperatively. His manifest refractive error in the right eye was -5.50 spherical diopters and astigmatism of -1.00 cylinder in the axis 50^o^ and in the left eye was -7.00 spherical diopters and astigmatism of -0.50 cylinder in the axis 90^o^. This patient is currently under observation.

## Discussion

We observed two cases of bilateral rhegmatogenous retinal detachment during orthokeratology treatment. Simultaneous bilateral rhegmatogenous retinal detachment is rare and is estimated to account for 1-2.5% of all retinal detachments [10,11]. The annual incidence of simultaneous bilateral retinal detachment is estimated to be 0.35 patients per 100,000 population [12]. Pediatric retinal detachment is also uncommon [13,14].

In our case, one patient was 16 years old, and the other was nine years old, both undergoing orthokeratology against their high myopia at different eye clinics. Severe myopia is a known risk for rhegmatogenous retinal detachment. Unfortunately, they were discovered after the retinal detachment had progressed beyond the fovea. Orthokeratology is a relatively safe treatment [15,16]. Some reports suggest that orthokeratology may prevent elongation of the axial length [1-3]; however, the risk of rhegmatogenous retinal detachment still remains. More to the point, wearing and removing hard contact lenses can irritate degenerative areas of the retina and induce the formation of retinal detachment, as is said to occur in patients with atopic dermatitis [17].

It is difficult to determine whether orthokeratology treatment is the cause of the retinal detachment in our cases. To the best of our knowledge, there are no case reports of bilateral retinal detachment during orthokeratology although there are many reports of sight-threatening complications in cornea and infection [4,5]. We believe it is unnatural that there have been many reports of anterior segment complications during orthokeratology treatment, but no reports of retinal detachment even in large surveys. It is possible that many cases of retinal detachment were not reported solely because of myopia and the relationship to orthokeratology treatment was not discussed. This could be due to improper use. Patients with atopic dermatitis often rub or scratch the periorbital area day and night. However, since patients during orthokeratology treatment only wear their contact lenses at night, it is expected to be difficult to determine if they are rubbing or scratching. With the implementation of large-scale surveys and the accumulation of case reports without dismissing the case as a simple retinal detachment due to myopia, the causal relationship between orthokeratology treatment and retinal detachment will gradually become clearer. Proactive repeat fundus examinations would at least provide an opportunity to detect retinal detachment at an early stage when vision is not affected.

It is often difficult to thoroughly examine the peripheral retina of young children. It is possible that the opticians and refractive ophthalmologists do not recognize the possibility of retinal detachment formation and do not adequately observe the retina, especially in the periphery, and it is concerning that other cases can be found in the future. Since orthokeratology is getting popular in developed countries [6,7], these cases should be brought to the widespread attention of not only opticians and refractive specialists but also retinal specialists.

## Conclusions

Orthokeratology is a good treatment for myopia correction, but it is not sufficient to overcome complications of high myopia such as retinal detachment. Because orthokeratology is often applied to young children who are not cooperative in fundus examinations and often do not complain of visual impairment if they can see out of one eye, it requires not only periodic anterior segment examinations but also more careful fundus examinations, especially of the peripheral retina. It is important for refractive specialists and optometrists to understand the presence of these cases and to collaborate with retina specialists.
